# A comparative study of the laboratory features of COVID‐19 and other viral pneumonias in the recovery stage

**DOI:** 10.1002/jcla.23483

**Published:** 2020-07-21

**Authors:** Guolian Zhao, Yingying Su, Xiaomeng Sun, Xiaoli Cui, Liyun Dang, Lijuan Zhao, Xiaowen Tan, Hongrui Wang, Ming Yang

**Affiliations:** ^1^ Department of Molecular Biology College of Basic Medical Sciences Jilin University Changchun China; ^2^ Department of Laboratory Medicine Xi'an Chest Hospital Xi’an China; ^3^ Department of Immunology College of Basic Medical Sciences Jilin University Changchun China; ^4^ The Director of Xi'an Chest Hospital Xi'an China

**Keywords:** COVID‐19, follow‐up, lymphocyte, neutrophil, recovery, SARS‐CoV‐2, total protein, viral pneumonia

## Abstract

**Background:**

Clinical recovery does not mean full recovery. It is necessary to explore the aftereffects of COVID‐19 in patients and compare the laboratory features of COVID‐19 and other viral pneumonias in the recovery stages.

**Methods:**

Forty‐seven cases of COVID‐19 and 45 cases of other viral pneumonias (control) were included in this study. The laboratory parameters were compared between COVID‐19 and control patients as well as severe and moderate COVID‐19 patients from the clinical recovery stage to the 4 weeks postdischarge recovery stage.

**Results:**

A higher RDW‐CV level and neutrophil percentage and lower levels of total proteins, lymphocytes, eosinophils, and MCH were found in COVID‐19 patients compared with those in controls from the clinical recovery to the postdischarge recovery stages. Further analysis showed that decreases in lymphocytes, total proteins, and SOD and elevations in neutrophils, FDP, CRP, and ESR were more common in severe than moderate cases of COVID‐19 during hospitalization; however, differences in these indicators, except total proteins, were not observed in the postdischarge recovery stages. Additionally, only 76.9% of COVID‐19 patients were positive for IgG antibodies against SARS‐CoV‐2 in the convalescence stage, and one patient that was negative for specific IgG was reinfected.

**Conclusions:**

This study demonstrated that patients recovering from COVID‐19 might need better care than that patients with other viral pneumonias due to the possibility of having poor immunity and nutritional conditions. These findings provide new insights to improve the understanding of COVID‐19 and improve care for patients affected by these kinds of pandemics in the future.

## Graphical abstract

A higher neutrophil percentage and lower levels of total proteins and lymphocytes were found in COVID‐19 patients compared with those in other viral pneumonia patients from the clinical recovery to the postdischarge recovery stages. This study demonstrated that patients recovering from COVID‐19 might need better care than that patients with other viral pneumonias due to the possibility of having poor immunity and nutritional conditions.

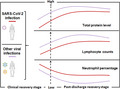

**Abbreviations**
ARDS: acute respiratory distress syndrome
COVID‐19: coronavirus disease 2019
CRP: C‐reactive protein
CT: computed tomography
ESR: erythrocyte sedimentation rate
FDP: fibrinogen degradation products
IQR: interquartile range
MCH: mean corpuscular hemoglobin
RDW‐CV: red blood cell distribution width‐correlation variance
rRT‐PCR: real‐time reverse transcription‐polymerase chain reaction
SARS‐CoV‐2: severe acute respiratory syndrome coronavirus 2
SOD: superoxide dismutase

## 1 INTRODUCTION

A novel coronavirus (SARS‐CoV‐2) is the etiological agent responsible for the ongoing pandemic of coronavirus disease 2019 (COVID‐19). The genome of the virus, which belongs to lineage B of the betacoronavirus genus, has nearly 80% similarity to the genome of the severe acute respiratory syndrome coronavirus (SARS‐CoV).^1^, ^2^ Infection with SARS‐CoV‐2 is more likely to affect older men with chronic illnesses and could result in acute respiratory distress syndrome (ARDS).^3^ Moreover, lymphopenia and inflammatory cytokine storms could be associated with disease severity and fatal outcomes.^4^ As of May 20, 2020, 4,947,929 cases have been confirmed worldwide, and 324,776 patients have died. Fortunately, more than 1.7 million people around the world are known to have already been discharged from the hospital, according to data from Johns Hopkins University. However, clinical recovery does not necessarily mean full recovery; some COVID‐19 patients still have a mild cough and feel tired even once they are considered recovered and are no longer contagious, and usually, it takes a long time for patients to feel fully normal.^5^

There are still many uncertainties regarding recovered patients with COVID‐19: How does the illness affect them in the long run, and what are the different features that distinguish COVID‐19 and other infectious diseases in the recovery stages? It is noteworthy that other viral pneumonias share similar epidemiological characteristics and clinical manifestations with COVID‐19 and occur in the same seasons.^6^ Currently, several studies have compared the differences in epidemiology, clinical manifestations, and laboratory characteristics between SARS‐CoV‐2–positive and SARS‐CoV‐2–negative patients on admission. As reported, urea, creatinine, and procalcitonin were important features discriminating COVID‐19 from SARS‐CoV‐2–negative patients.^7^ Additionally, COVID‐19 patients were more likely to exhibit a nonproductive cough, fatigue, gastrointestinal symptoms, and ground‐glass opacities than H1N1 patients.^8^ However, few reports have focused on the characteristics of COVID‐19 patients after discharge upon follow‐up, and the differences between COVID‐19 and other viral pneumonias in the recovery stages remain to be elucidated. In this study, a systematic review and pooled analysis were performed to compare the laboratory characteristics of COVID‐19 patients and patients with other viral pneumonias from the clinical recovery stage to the 4 weeks postdischarge recovery stage. It is hoped that these findings will provide additional useful and supplementary information for understanding COVID‐19 and for improving therapies and care for patients in similar kinds of pandemics in the future.

## 2 METHODS

### 2.1 Study participants

For this retrospective, single‐center study, 47 patients with COVID‐19 and 45 patients with other viral pneumonias (control) were included in this study. All patients were confirmed by laboratory tests and were hospitalized at Xi'an Chest Hospital (Shaanxi Province of China) from January 31 to April 3, 2020. A patient can be confirmed as having COVID‐19 if they are positive based on either a nucleic acid test or serum anti–SARS‐CoV‐2 IgM detection based on the 7th edition of the guidelines provided by the National Health Commission of China. The 47 cases of COVID‐19 were divided into 32 moderate cases and 15 severe cases patients based on the diagnostic criteria recommended by the Chinese National Institute for Viral Disease Control and Prevention. Briefly, moderate patients had symptoms such as fever and respiratory tract symptoms, and imaging showed pneumonia, while severe patients had respiratory distress (RR ≥ 30 beats/minute in a resting state) or a mean oxygen saturation of ≤93% (an arterial blood oxygen partial pressure (PaO2)/oxygen concentration (FiO2) ≤300 mm Hg). In the 45 cases of the control group, patients infected with influenza B (17 cases), influenza A (12 cases), respiratory syncytial virus (8 cases), parainfluenza virus (6 cases), and adenovirus (2 cases) were confirmed by PNEUMOSLIDE IgM serological tests, and SARS‐CoV‐2 infection was excluded by nucleic acid and antibody tests. The study was approved by the Ethics Committees of Xi'an Chest Hospital.

### 2.2 Data collection

Demographic features, clinical symptoms, and laboratory results were obtained from electronic medical records. Usually, the median time from onset to clinical recovery for mild cases is approximately 2 weeks and is 3‐6 weeks for patients with severe disease. COVID‐19 patients in clinical recovery should meet the following criteria: normal body temperature for more than three days, two negative RT‐PCR tests of respiratory specimens at 24‐hour intervals, and a chest CT (computed tomography) showing that the lesion is essentially absorbed or that only a few fibrous stripes can be observed. The laboratory results, including hematological and biochemical data, were collected at the time of admission and at different recovery stages. In broad terms, the recovery stages included the clinical recovery stage (1‐3 days before discharge) and the postdischarge recovery stages (2‐ and 4‐week follow‐up visits). The collected data were independently reviewed and checked by two reviewers.

### 2.3 Laboratory measurements

Real‐time reverse transcription‐polymerase chain reaction (rRT‐PCR) was used for the SARS‐CoV‐2 nucleic acid test. Briefly, throat swab samples were collected for extracting viral RNA from patients. Then, the rRT‐PCR assay was performed using a SARS‐CoV‐2 nucleic acid detection kit according to the manufacturer's protocol (ShengXiang Biotech Co Ltd). The IgM and IgG antibodies against SARS‐CoV‐2 in serum samples were tested using colloidal gold immunochromatography assay kits supplied by Lizhu Reagent Co., Ltd. A PNEUMOSLIDE kit (Vircell) was employed to detect IgM antibodies against 9 common respiratory pathogens, including *Legionella pneumophila, Mycoplasma pneumoniae, Coxiella burnetii, Chlamydia pneumoniae*, adenovirus, respiratory syncytial virus, influenza A virus, influenza B virus, and parainfluenza virus types 1, 2, and 3.

The clinical laboratory measurement results, including serum biochemical, routine blood, and blood coagulation test results, were collected during routine clinical practice. The BC‐6800plus automated blood analyzer (Mindray Medical International Co., Ltd) and ADVIA 2400 automatic chemical analyzer (Bayer) were used to analyze routine blood and biochemical parameters, respectively. Evaluation of blood coagulation was performed by an ACL TOP 700 (Werfen). All laboratory parameters were obtained via standard automated laboratory methods by using the appropriate commercially available kits according to the manufacturer's protocols. Additionally, the numbers of total T, CD4^+^ T, CD8^+^ T, NK, and B cells were analyzed in the patients in the SARS‐CoV‐2–positive and control groups by flow cytometry. The antibodies against cell surface molecules were purchased from the BD Company. All samples were detected and analyzed by a BD FACSCanto II Flow Cytometry System.

### 2.4 Statistical analysis

All laboratory statistical data are presented as the mean ± SEM; age and number of days are described as the median (interquartile range values, IQR), and categorical variables are described as the number (percentage). Independent group *t* tests or Mann‐Whitney tests were used to compare means. Chi‐squared and Fisher's exact tests were used to compare proportions for categorical variables. Two‐sided comparisons with a p value less than 0.05 were considered significant. The data were analyzed using SPSS 16 (Chicago, USA) and GraphPad Prism 8.0.

## 3 RESULTS

### 3.1 Clinical and laboratory characteristics of COVID‐19 and other viral pneumonia patients at admission

The demographics and clinical manifestations of 47 COVID‐19 patients and 45 patients with other viral pneumonias (control) are summarized in Table 1. As shown, the median age of COVID‐19 patients was 52 years, which was older than that of the control patients (42 years) but without a significant difference. Meanwhile, no obvious differences were found in terms of the gender distribution or major clinical manifestations between the two groups, except that diarrhea was more common and expectoration was less common in COVID‐19 patients. On the other hand, the severe COVID‐19 group had more patients with high fever (>39℃) and an older median age (62 years vs 48 years) compared with the moderate group (*P* < .05).

**Table 1:** Clinical baseline characteristics of COVID‐19 patients and control cases at admission

	Control patients (45)	COVID‐19 patients (47)	*P* value	Moderate COVID‐19 cases (32)	severe COVID‐19 cases (15)	*P* value
Characteristics						
Median age (IQR, yrs)	42 (31‐56.5)	52 (35‐63)	.060	48 (31.5‐57)	62 (51‐72)	.002
Gender:						
Male (%)	24 (53.3%)	19 (40.4%)	.215	14 (43.8%)	5 (33.3%)	.498
Female (%)	21 (46.7%)	28 (59.6%)		18 (56.3%)	10 (66.7%)	
Signs and symptoms						
Fever (%)	35 (77.8%)	37 (78.7%)	.912	23 (71.9%)	14(93.3%)	.196
Highest temperature (℃)						
37.3‐38.0 (%)	14 (31.1%)	19 (40.4%)	.272	12 (37.5%)	7 (46.7%)	.010
38.1‐39.0 (%)	15 (33.3%)	13 (27.7%)		10 (31.3%)	3 (20.0%)	
39.0‐40.0 (%)	6 (13.3%)	5 (10.6%)		1 (3.1%)	4 (26.7%)	
Cough (%)	33 (73.3%)	25 (53.2%)	.054	14 (43.8%)	11 (73.3%)	.058
Expectoration (%)	23 (51.1%)	11 (23.4%)	.006	6 (18.8%)	5 (33.3%)	.465
Pharyngalgia (%)	2 (4.4%)	5 (10.6%)	.467	4 (12.5%)	1 (6.7%)	.923
Chest tightness (%)	7 (15.6%)	7 (14.9%)	.930	6 (18.8%)	1 (6.7%)	.519
Diarrhea (%)	0 (0.0%)	7 (14.9%)	.021	5 (15.6%)	2 (13.3%)	1.000
Headache (%)	3 (6.7%)	2 (4.3%)	.960	2 (6.3%)	0 (0.0%)	1.000
Myalgia (%)	2 (4.4%)	5 (10.6%)	.467	3 (9.4%)	2 (13.3%)	1.000
Fatigue (%)	5 (11.1%)	12 (25.5%)	.075	9 (28.1%)	3 (20.0%)	.813
Short of breath (%)	3 (6.7%)	3 (6.4%)	1.000	1 (3.1%)	2 (13.3%)	.487
Hospitalization (IQR, d)	11 (9‐15.5)	17 (15‐21)	.000	17 (11.25‐21)	19 (17‐22)	.069

Note

*P* < .05 was considered statistically significant.

Abbreviation: IQR, interquartile range.

The major differences in laboratory findings between COVID‐19 and control patients at admission are shown in Table 2. Higher values for the red blood cell distribution width‐correlation variance (RDW‐CV, *P* < .01) and lower levels of total protein (*P* < .05), whole blood cells (*P* < .05), basophils (*P* < .01), and eosinophils (*P* < .01) appeared in COVID‐19 patients compared with those in control cases. Moreover, more patients with hypoalbuminemia (serum albumin ≤ 40 g/L) and lymphopenia (lymphocyte number ≤ 1.1 × 10^9^/L) were observed in the COVID‐19 group as well (*P* < .05). Furthermore, the circulating immune cell subsets in COVID‐19 patients were analyzed. The results demonstrated that the numbers of total T lymphocytes (*P* < .05), CD4^+^ T cells (*P* < .01), B cells (*P* < .05), and NK cells (*P* < .05) in COVID‐19 patients were reduced significantly compared with those in control patients.

**Table 2:** Laboratory parameters in COVID‐19 and control patients at admission

Variants (Normal range)	Control patients (45)	COVID‐19 patients (47)	*P* value
Albumin (40‐55 g/L) (Mean ± SE)	41.7 ± 3.8	40 ± 4.4	.062
<40 (n, %)	11 (24.4%)	21 (44.7%)	.042
≥40 (n, %)	34 (75.6%)	26 (55.3%)	
Total protein (65‐85 g/L)	68.2 ± 4.6	64.9 ± 5.3	.002
Whole blood cell (4.0‐10.0 × 10^9^/L)	6.25 ± 2.76	5.16 ± 1.79	.030
Monocyte (0.1‐0.6 × 10^9^/L)	0.46 ± 0.22	0.40 ± 0.20	.193
Red blood cell (3.8‐5.1 × 10^9^/L)	4.28 ± 0.66	4.52 ± 0.81	.135
Red blood cell distribution width‐CV (11%‐16%)	12.24 ± 0.62	13.10 ± 1.62	.002
Lymphocyte (1.1‐3.2 × 10^9^/L)	1.49 ± 0.55	1.30 ± 0.58	.112
≤1.1	9 (20.0%)	21 (44.7%)	.012
>1.1	36 (80.0%)	26 (55.3%)	
Basophils (0‐0.1 × 10^9^/L)	0.014 ± 0.008	0.008 ± 0.009	.002
Eosinophils (0.02‐0.52 × 10^9^/L)	0.085 ± 0.079	0.034 ± 0.051	.000
Neutrophils (2.0‐6.0 × 10^9^/L)	4.20 ± 2.60	3.56 ± 1.79	.178
Neutrophil percentage (50%‐70%)	63.5 ± 13.5	65.1 ± 12.3	.543
T lymphocytes (×10^6^/L)	1257.1 ± 476.1	989.4 ± 459.7	.027
T lymphocytes (%)	73.3 ± 9.3	72.1 ± 10.1	.605
CD4^+^ T cells (×10^6^/L)	755.6 ± 337.4	512.0 ± 276.5	.003
CD4^+^ T cells (%)	42.4 ± 9.0	37.6 ± 12.1	.078
B cells (×10^6^/L)	230.9 ± 110.5	154.9 ± 71.5	.012
B cells (%)	13.2 ± 4.4	12.3 ± 5.8	.625
NK cells (×10^6^/L)	277.6 ± 143.4	197.7 ± 100.6	.045
NK cells (%)	16.3 ± 8.0	14.7 ± 9.9	.607

Note

*P* values indicate differences between COVID‐19 and control patients. *P* < .05 was considered statistically significant.

### 3.2 Differences in laboratory parameters in COVID‐19 and control patients in the recovery stages

**Figure 1:**
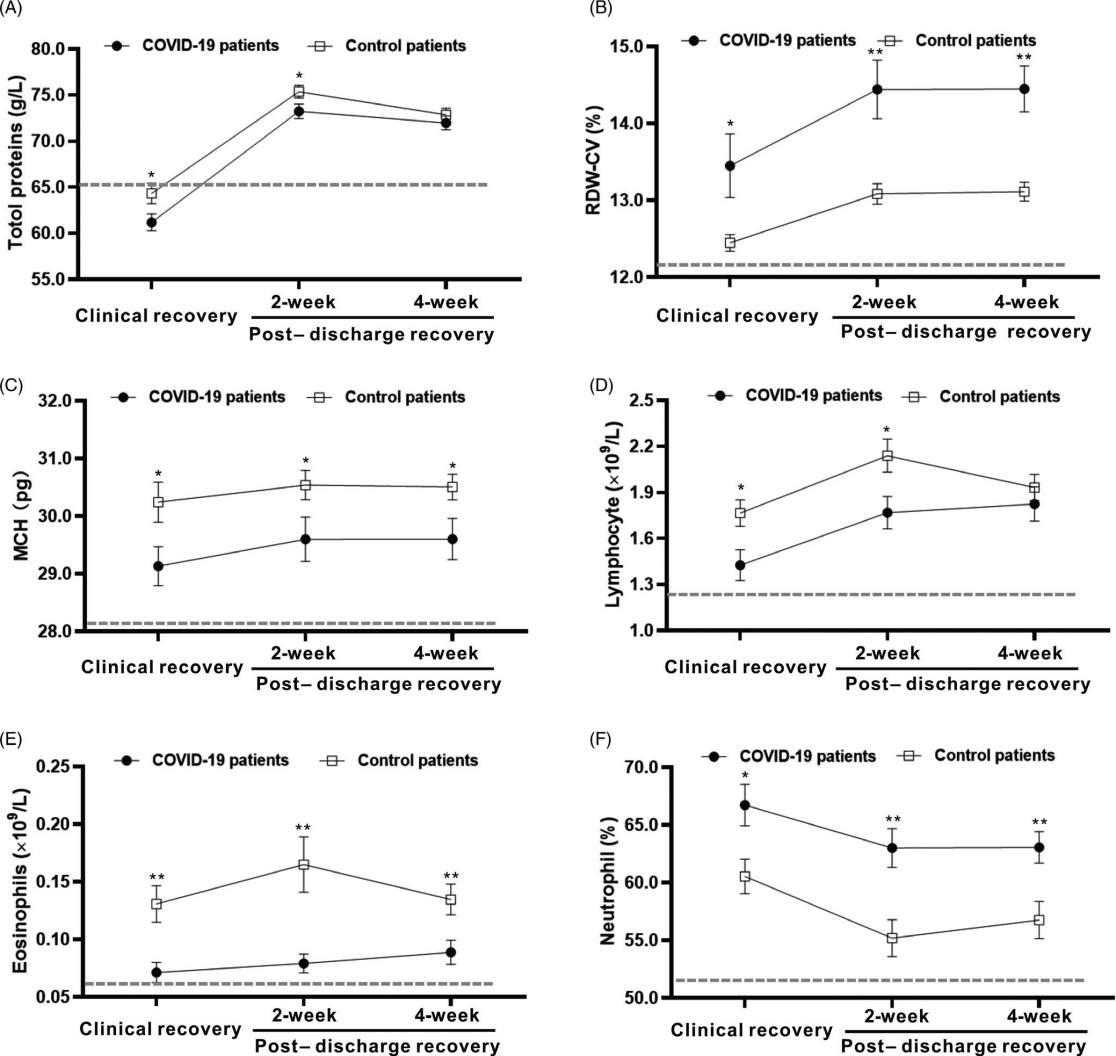
Dynamic profile of the laboratory parameters in COVID‐19 and control patients in the recovery stages. Timeline charts illustrate the laboratory parameters in COVID‐19 and control patients from the clinical recovery stage to the 4 weeks postdischarge recovery stage. The dashed lines in gray show the lower normal limit of each parameter. A. Total proteins; B. red blood cell distribution width‐correlation variance (RDW‐CV); C. mean corpuscular hemoglobin (MCH); D. lymphocytes; E. eosinophils; and F. neutrophil percentage. * indicates *P* < .05 for COVID‐19 patients vs control patients at a single point; ** indicates *P* < .01 for COVID‐19 patients vs control patients at a single point

To observe the major differences between COVID‐19 and other viral pneumonias in the recovery stages, the dynamic profiles of the major laboratory parameters in COVID‐19 and control patients were tracked from the clinical recovery stage to the 4 weeks postdischarge recovery stage. As shown in Figure 1, we discovered that COVID‐19 patients showed higher values for the RDW‐CV (*P* < .01), neutrophil percentage (*P* < .05), and prolonged prothrombin time (PT, *P* < .05) compared with the control patients, and they also showed lower levels of total proteins (*P* < .05), lymphocytes (*P* < .05), eosinophils (*P* < .01), and mean corpuscular hemoglobin (MCH, *P* < .05) at the clinical recovery stage compared with the control patients. As recovery continued, comparable levels of total proteins and lymphocytes were observed between COVID‐19 and control patients at the 4‐week follow‐up visit after discharge. However, differences in the RDW‐CV, neutrophil percentage, eosinophil, and MCH values still existed in COVID‐19 patients compared with those in control patients at all recovery stages (Figure 1). These findings indicated that decreased antiviral immunity, poorer nutritional conditions, and an increased inflammatory response potentially existed in COVID‐19 patients compared with patients with other viral pneumonias in the recovery stages.

### 3.3 Differences in laboratory parameters in severe and moderate COVID‐19 patients in the recovery stages

To further investigate the effect of the severity of COVID‐19 in the recovery stages, the dynamic laboratory parameters in severe and moderate COVID‐19 patients were compared as well. At the clinical recovery stage, severe patients with COVID‐19 had lower levels of total proteins (*P* < .05), albumin (*P* < .01), superoxide dismutase (SOD, *P* < .01), and lymphocytes (*P* < .01); higher levels of neutrophils (*P* < .05), C‐reactive protein (CRP, *P* < .01), and fibrinogen degradation products (FDP, *P* < .05); and a higher erythrocyte sedimentation rate (ESR, *P* < .01) than moderate patients (Table 3). However, differences in these indicators, except for total proteins (70.7 ± 5.2 vs 74.3 ± 4.1 g/L, *P* < .05), were not observed in the postdischarge recovery stages.

**Table 3:** Laboratory parameters in severe and moderate COVID‐19 patients at clinical recovery stage

Variants (Normal range)	Moderate COVID‐19 cases (32)	Severe COVID‐19 cases (15)	*P* value
Albumin (40‐55 g/L) (Mean ± SE)	39.5 ± 4.1^a^	34.6 ± 4.1 ^a^	.001
Total protein (65‐85 g/L)	62.6 ± 5.6 ^a^	57.8 ± 6.4 ^a^	.018
C‐reactive protein (0‐6 mg/L)	5.3 ± 10.3	39.6 ± 56.9 ^b^	.003
Superoxide dismutase (129‐216 U/L)	165.1 ± 17.1	135.4 ± 18.4	.002
Fibrinogen degradation products (0‐5 μg/mL)	2.0 ± 1.9	4.3 ± 3.9	.026
Erythrocyte sedimentation rate (0‐15 mm/h)	32.7 ± 27.7 ^b^	63.4 ± 34.3 ^b^	.005
Lymphocyte (1.1‐3.2 × 10^9^/L)	1.63 ± 0.67	1.00 ± 0.53 ^a^	.002
Neutrophils (2.0‐6.0 × 10^9^/L)	3.85 ± 1.46	5.47 ± 2.89	.014

Note

*P* values indicate differences between severe and moderate COVID‐19 patients. *P* < .05 was considered statistically significant.

^a^ Indicates the values lower than the normal level of each parameter.

^b^ Indicates the values higher than the normal level of each parameter.

### 3.4 Antibody responses of convalescence stage patients with COVID‐19

We evaluated the specific IgM and IgG antibody responses against SARS‐CoV‐2 in convalescent serum samples from 26 COVID‐19 patients at their 4‐week follow‐up visit. The results showed that positive IgG antibodies were detected in 20 patients (76.9%) (Table 4). Only 15 patients were positive for IgM antibody detection, since too long a time had elapsed from the onset of illness to 4 weeks after discharge. Collectively, 4 patients (15.4%) were double‐negative for IgG and IgM detection (Table 4), and one of them was confirmed to have been reinfected in the convalescent phase.

**Table 4:** Detection of IgM and IgG seropositivity for COVID‐19 patients at the 4‐wk follow‐up visit

	IgM against SARS‐CoV‐2 (n, %)	IgG against SARS‐CoV‐2 (n, %)	Both IgM and IgG against SARS‐CoV‐2 (n, %)
Positive	15 (57.7%)	20 (76.9%)	15 (57.7%)
Negative	11 (42.3%)	6 (23.1%)	4 (15.4%)

## 4 DISCUSSION

Currently, over one‐third of COVID‐19 patients in the world have recovered and been discharged after infection and treatment. However, discharge from the hospital should not be considered the endpoint of monitoring and precautionary measures.^9^ For COVID‐19 patients, especially severe and critically ill patients, the road to full recovery could still be lengthy. Moreover, it is necessary to evaluate the possibility of reinfection in patients recovering from SARS‐CoV‐2. Therefore, regular follow‐up visits should be conducted for recovered COVID‐19 patients in the convalescent phase, which would be helpful to evaluate any changes in the acquired immune function, blood parameters, and biochemical factors and to monitor their health status to detect any possible future complications.

In this study, we evaluated the specific antibody response against SARS‐CoV‐2 by using convalescent serum samples obtained at the 4‐week follow‐up visit. Additionally, we compared the laboratory results of patients with COVID‐19 and other viral pneumonias (control) from the clinical recovery stage to the 4 weeks postdischarge recovery stage. Among the recovered COVID‐19 patients, 15.4% were double‐negative for specific IgM and IgG antibodies against SARS‐CoV‐2, and one patient was reported to have been reinfected in the recovery phase. This finding confirmed that not all recovered COVID‐19 patients developed specific antibodies. Additionally, higher red blood cell distribution width‐CV (RDW‐CV) and neutrophil percentage values as well as lower levels of total protein, prothrombin time, lymphocytes, eosinophils, and mean corpuscular hemoglobin (MCH) were found in the COVID‐19 patients than in the control patients in the recovery stages. Knowing the laboratory features and aftereffects of recovered patients will be helpful to ascertain future disease complications and will provide more information for the improvement of therapies and care for patients affected by these kinds of pandemics in the future.

In terms of laboratory findings, the patients with COVID‐19 had lower levels of total proteins and lymphocytes compared with the control patients during hospitalization and in the 2 weeks postdischarge recovery stage. Protein levels are usually used to evaluate the nutritional condition of patients.^10^ The decreased level of protein reflected a high consumption state in terms of nutrition, which suggested that COVID‐19 patients needed additional nutrition during hospitalization and even in the recovery stages compared with the control patients. Lymphocytes play a decisive role in maintaining immune homeostasis and the inflammatory response throughout the body, and a reduction in lymphocytes could result in reduced immunity.^11^ In this study, we observed pronounced lymphopenia with low total T‐ and B‐lymphocyte counts in COVID‐19 patients compared with that in control patients at admission, which was consistent with the result of a recent cohort study regarding the differences between COVID‐19 and H1N1 infection.^8^ The potential reasons for greater lymphocyte deficiency in COVID‐19 patients are as follows. (a) Since lymphocytes express the coronavirus receptor ACE2, SARS‐CoV‐2 can directly infect lymphocytes to destroy lymphatic organs, resulting in lymphocyte death.^12^ (b) Increases in inflammatory cytokines induced by SARS‐CoV‐2 may lead to increased lymphocyte apoptosis compared with that induced by other viruses.^13^ T cells, especially CD4^+^ T cells, were the most remarkably decreased subset in COVID‐19 patients compared with the control patients at admission. Since CD4^+^ T cells are critical for the regulation of both cellular immunity and humoral immunity, it is reasonable that these cells are the most sensitive to the total antivirus immune response.^12^, ^13^ Moreover, B cells were also found at lower levels in COVID‐19 patients than in control patients. B cells are responsible for specific antibody production against invaders, and a decrease in the response of B cells could result in a failure to restrict virus expansion and release.^14^ The reduction in lymphocytes may eventually diminish host antiviral immunity, which promotes infection. Therefore, lymphocyte activation treatments may be considered for recovered COVID‐19 patients and could be helpful to compensate for the potential dysfunction of the adaptive immune system.

It has been reported that increases in inflammatory mediators play a crucial role in fatal pneumonia caused by pathogenic human coronaviruses, including SARS‐CoV‐2.^15^ Neutrophils are considered to play an active role in inflammation. A higher neutrophil percentage was found in COVID‐19 patients than in control patients at the point of clinical recovery and even 4 weeks after discharge. We speculated that there were potential mechanisms underlying the increases in neutrophil levels caused by SARS‐CoV‐2 infection. (a) Coronavirus binding proteins might affect neutrophils or other related classic inflammatory mechanisms. Two neutrophil‐enriched genes, ANPEP and CEACAM1, are coronavirus receptors, indicating that neutrophils could be recruited by SARS‐CoV‐2.^16^ (b) Lung epithelial cells overexpress 6 classic neutrophil chemokines, CXCL1, CXCL2, CXCL3, CXCL5, CXCL8, and CXCL20, after SARS‐CoV‐2 infection.^16^ (c) SARS‐CoV‐2–infected lung cells also overexpressed complement C3 and associated pathway activation genes, while the receptor for C3a anaphylatoxin is a “neutrophil degranulation” gene; C3 and complement activation has been recently shown to be involved in ARDS with systemic inflammation.^17^ (d) A high level of tumor necrosis factor (TNF) was found in COVID‐19 patients, and it plays a well‐established role in neutrophil activation and prolongs neutrophil survival.^18^ Similarly, another long‐term elevated indicator in COVID‐19 patients was RDW‐CV, which reflects the variation in the size of RBCs and has been reported to be correlated with critical diseases, including acute exacerbation of interstitial pneumonia and ARDS.^19^ A possible explanation for its elevation is the strengthening of the pro‐inflammatory state, resulting in the structural and functional alteration of RBCs.^20^ Relatively lower levels of eosinophils were found in the COVID‐19 group than in the control group during the hospitalization and postdischarge recovery stages. The reduction in eosinophils might be related to a mechanism associated with the stress response in acute lung injury caused by SARS‐CoV‐2, which may inhibit the release of eosinophils in the bone marrow through glucocorticoid secretion.^21^ Taken together, the differences in these indicators reflect the presence of a potential contiguous inflammatory condition in recovered COVID‐19 patients that may not affect patients with other viral pneumonias. This suggests that it might be necessary to target specific inflammatory mechanisms in the early stage of COVID‐19.

Decreases in the lymphocyte counts and total proteins and increases in neutrophils were more common in severe cases than in moderate cases of COVID‐19 during hospitalization. In addition to the above indicators, lower levels of superoxide dismutase (SOD), higher levels of fibrinogen degradation products (FDP) and C‐reactive protein (CRP), and a higher erythrocyte sedimentation rate (ESR) were also found in severe COVID‐19 patients in the clinical recovery stage. SOD is one of the major enzymes in the antioxidant defense system. The pathogenesis of virus infection is usually related to oxidative stress. A previous study ^22^ reported that H5N1 infection in lung epithelial cells decreased SOD expression at the mRNA and protein levels. Decreased SOD expression could significantly enhance the production of reactive oxygen species and increase the pro‐inflammatory response. Generally, an excessive inflammatory response is associated with the severity of SARS‐CoV‐2 infection.^15^ High levels of two inflammatory markers, CRP and ESR, were found to be associated with the severity of COVID‐19 during hospitalization in this study, thus confirming earlier results.^23^, ^24^ Similar findings emerged for FDP and PT, which were confirmed by a previous report to be moderately or markedly elevated in all cases of COVID‐19 involving death.^25^, ^26^ Interestingly, differences in these indicators were not observed between severe and moderate COVID‐19 patients in the postdischarge recovery stages, except for total proteins. This result suggested that the decreased lymphocyte counts and increase in the inflammatory response in COVID‐19 patients might not only be caused by the level of severity compared with other viral pneumonia cases but may also depend on the specific characteristics of coronavirus.

Dynamic laboratory data observation from clinical recovery to the postdischarge stage is more informative than observation at a single time point and contributes to more accurate laboratory analysis of COVID‐19. In conclusion, poor immunity and nutritional conditions as well as a potential increase in the inflammatory response might play a larger role in the disease course in COVID‐19 patients than in patients with other viral pneumonias in the recovery stages. These findings provide new insights for improving the understanding of the complexities of SARS‐CoV‐2 pathogenesis and improving therapies and care for patients affected by these kinds of pandemics in the future.

## AUTHOR CONTRIBUTIONS

MY designed the study and drafted the article. GLZ and YYS collected and analyzed the data. XLC and XWT reviewed the data. XMS and LJZ contributed to the statistical analysis. LYD took responsibility for obtaining ethical approval. HRW and YYS revised the article.
